# Association of Preoperative Opioid Use and Postoperative Opioid Requirement in Revision Total Knee Arthroplasty

**DOI:** 10.7759/cureus.76812

**Published:** 2025-01-02

**Authors:** Zachary C Lum, Christopher T Holland, Daniel T O'Connor, Arta Gharib-Parsa, Ana Barragan-Trejo, Jeannie Y Park, Mauro Giordani, John P Meehan

**Affiliations:** 1 Orthopedic Surgery, University of California (UC) Davis School of Medicine, Sacramento, USA; 2 Orthopedic Surgery, Nova Southeastern University, Fort Lauderdale, USA; 3 Orthopedic Surgery, Campbell Clinic, Durham, USA

**Keywords:** morphine milligram equivalent, narcotic, opioid crisis, opioids, total knee revision arthroplasty

## Abstract

Purpose

The characterization of opioid usage in revision total knee arthroplasty (rTKA) remains incomplete. This study aimed to evaluate postoperative opioid consumption, measured in morphine milligram equivalents (MME), as well as hospital length of stay and discharge destinations such as home versus skilled nursing facility following revision TKA.

Methods

Patients who underwent rTKA between 2010 and 2018 were assessed for preoperative opioid use and monitored for 24 months postoperatively. Patients were stratified into opioid-naïve or opioid-tolerant categories. Demographic data were collected, and opioid prescriptions and average MME were compared between the two groups.

Results

Out of 173 patients who underwent rTKA, 91 (53%) were categorized as opioid-tolerant, with an average preoperative MME of 23.5 mg/day. Postoperatively, opioid-tolerant patients exhibited higher daily MME at 3 and 6 months and were more likely to receive opioid prescriptions at 6 and 12 months. Additionally, the opioid-tolerant group experienced a significantly prolonged postoperative hospital stay at 4.82 days versus 3.78 days (p=0.004) and were more inclined to be discharged to a skilled nursing facility (rather than home) compared to the opioid-naïve group, at 40.7% versus 18.3% (p=0.004).

Conclusions

Opioid-tolerant patients demonstrated elevated postoperative MME requirements, longer recovery periods, and an increased likelihood of skilled nursing facility discharge, underscoring the challenges associated with opioid tolerance in the post-rTKA setting. Both groups showed a reduction in opioid usage at 3 months postoperatively, reaching a plateau at 6 months. These insights can inform revision surgeons in patient counseling and managing expectations.

## Introduction

As the number of primary total knee arthroplasty (TKA) procedures continues to rise, driven by factors such as an aging population and increasing rates of osteoarthritis, a concomitant increase in the need for revision surgeries is observed [1]. Revision TKA (rTKA) poses a distinct set of challenges compared to primary TKA, primarily due to factors such as compromised bone quality, altered anatomy, and scar tissue from previous surgeries. These factors contribute to a higher risk of complications, including, but not limited to, infection, bleeding, nerve or blood vessel injury, and, in severe cases, mortality [2-3].

In recent years, the long-term use of opioids following knee arthroplasty has emerged as a significant concern within the medical and surgical community. Studies have indicated that prolonged opioid use post-surgery is associated with several adverse outcomes, including an elevated risk of requiring revision surgery, poorer functional recovery, and an increased likelihood of experiencing complications during the recovery process [4-6]. Despite the well-documented risks and adverse effects associated with opioid use in primary TKA, there remains a notable gap in the literature regarding opioid consumption patterns both before and after rTKA procedures.

Due to this, our study goals are to provide valuable insights into the patterns of opioid utilization in patients undergoing rTKA at our institution. By examining opioid consumption trends across the preoperative, perioperative, and postoperative periods, spanning a comprehensive two-year recovery period, we aim to understand and highlight opioid use in this specific patient population. Additionally, our investigation seeks to compare various clinical parameters, such as opioid morphine milligram equivalents (MME) and postoperative outcomes, between patients with no prior opioid exposure (opioid-naïve) and those with a history of opioid tolerance. Through this analysis, we aim to contribute to a deeper understanding of opioid management strategies in the context of rTKA, which may guide clinical practice and optimize patient care.

## Materials and methods

Following approval from the Institutional Review Board of the university, a retrospective analysis was conducted on 173 patients who underwent rTKA at a single academic quaternary care center between January 1, 2010, and September 1, 2018. Patients were identified retrospectively using the Current Procedural Terminology (CPT) code 27487, which specifically denotes the revision of total knee arthroplasty involving both the femoral and tibial components. Inclusion criteria encompassed patients aged 18 and above, without regard to their underlying diagnosis, type of anesthesia, BMI, or American Society of Anesthesiologists (ASA) score. However, patients who underwent single-component exchange (polyethylene swap) were excluded from the analysis. This exclusion was due to the variability of single-component exchange, and the diagnoses associated with this operation, such as infection or aseptic loosening, and the variable invasiveness of the procedure, which can range from patellar revision to polyethylene exchange to tibial component exchange. Data collection and methods protocol was similar to another study by Lum ZC et al. [7].

Demographic data, including age, sex, height, weight, BMI, and date of birth, were collected for each patient. A comprehensive chart review was conducted, documenting the date of surgery and any opioid medications prescribed to the patient within the three months preceding the rTKA surgery. Subsequently, the patient's medical records were systematically monitored for an additional 24 months postoperatively, with any subsequent opioid prescriptions meticulously recorded. All opioid medications were identified, quantified, and standardized into oral MME to facilitate comparison. Patients were then categorized as either opioid-naïve or pre-operative opioid use (POU) based on whether they had received a narcotic prescription in the three months leading up to the surgery. The total MME was then organized into three-month intervals for all patients and subjected to comparative analysis between the opioid-naïve and POU groups.

Consistent perioperative protocols were applied across all rTKAs. Patients underwent surgery utilizing a medial parapatellar approach. The specific revision technique employed was left to the discretion of the operating surgeon. Each surgeon was fellowship-trained in adult reconstruction knee replacement surgery, employed at the same academic university hospital, and had a minimum of 10 years of practice experience. Upon completion of the procedure, a multimodal analgesia protocol was uniformly administered to all patients to manage postoperative pain effectively. Patients were cared for in the orthopedic ward by nursing staff trained to care for orthopedic patients. Patients received acetaminophen, a non-steroidal anti-inflammatory such as celecoxib, a gabapentinoid such as gabapentin, and oxycodone. Patients who were allergic to oxycodone received hydrocodone and acetaminophen, ensuring that acetaminophen doses did not exceed 4 grams per day. Patients were encouraged to use ice packs. Additionally, for venothromboembolism prophylaxis, either enoxaparin or warfarin was employed, with a standard duration of six weeks.

Patients were scheduled for follow-up appointments on postoperative days 10-14, day 42, day 90, and subsequently on an annual basis. Depending on the individual's recovery progress, more frequent follow-up visits were arranged as needed. For patients experiencing persistent pain, a mechanism was in place for requesting refill prescriptions by reaching out to the nursing staff, who facilitated the process by obtaining approval from a physician. While medication refills typically took place during in-person postoperative visits, occasional requests were accommodated through telephone communication or the patient online portal.

Statistical analysis

The Student’s t-test was used to compare quantitative parametric variables, such as oral MME and length of stay between opioid-naïve and POU groups. Categorical non-parametric variables, such as patients still on opioids, were compared between groups using the Chi-squared test, as sample sizes were greater than 5. To account for multiple comparisons, a Bonferroni correction was applied, adjusting the significance level to reduce the risk of Type I errors.

## Results

Between 2010 and 2018, a total of 173 patients underwent two-component rTKA. Among them, 91 out of 173 (53%) were classified into the pre-operative opioid use group, with an average preoperative MME of 23.5 mg/day. Notably, factors such as patient age, duration of surgery, BMI, presence of diabetes, and functional status did not exhibit significant differences between the opioid-naïve and pre-op opioid groups (p > 0.05), as seen in Table 1.

**Table 1:** Preoperative patient demographics: Patient age, surgery duration, BMI, diabetes, and functional status were not significantly different between the naïve and pre-operative opioid use groups (p > 0.05). There was a trend for more females to be in the pre-operative opioid use group. ASA: American Society of Anesthesiologists scale; d: day.

	Opioid Naïve N=82	Pre-operative Opioid Use N=91	P-value
Age	64.8 years	65.6 years	0.85
Gender	46 female	64 female	0.06
BMI kg/m^3^	32.1	33.4	0.56
Duration of Surgery (minutes)	188.4	194.3	0.71
Smoker	9.6%	12.3%	0.55
Length of Stay (d)	3.78	4.82	0.004
ASA 1	0	0	0.63
ASA 2	40	38	
ASA 3	40	51	
ASA 4	2	2	

In the opioid-naïve group, the average daily MME postoperatively decreased from 21.6 mg/day in the first 3 months to 4.9 mg/day at 6 months, further declining to 4.6 mg/day at 12 months, and ultimately reaching 3.0 mg/day at 24 months. Notably, at 3-6 months postoperatively, 14 out of 82 patients (17%) were prescribed opioids, while at 12 months, 19 out of 82 patients (23%) were still prescribed opioids. By the 24-month mark, this number had slightly increased to 21 out of 82 patients (26%), as summarized in Table 2.

**Table 2:** Opioid-naïve and pre-op opioid use patients and their overall morphine milligram equivalents (MME) over a 27-month period. There were 82 patients in the naïve group and 91 in the POU group. Patients in the POU cohort had higher average MMEs for the first 6 months and were more likely to require opioids at the 6- and 12-month time points. POU: Preoperative opioid use.

Time Interval	Opioid Naïve			Pre-operative Opioid Use			P-values	
	# taking opioids	% taking opioids	Avg MME (mg/day)	# taking opioids	% taking opioids	Avg MME (mg/day)	# of patients taking opioids (pre-op opioid v naïve)	Avg MME (mg/day) (preop opioid v naïve)
3 months Pre Op	0	0%	-	91	100%	23.5	-	-
0-3 months Post Op	80	97.5%	21.6	88	96.7%	31.2	p=0.49	p<0.01*
3-6 months Post Op	14	17.1%	4.9	34	37.4%	11.9	p<0.002*	p<0.0019*
6-12 months Post Op	19	23.1%	4.6	33	36.3%	7.6	p<0.04*	p=0.06
12-24 months Post Op	21	25.6%	3.04	33	36.3%	6.79	p=0.8	p=0.12

**Figure 1:**
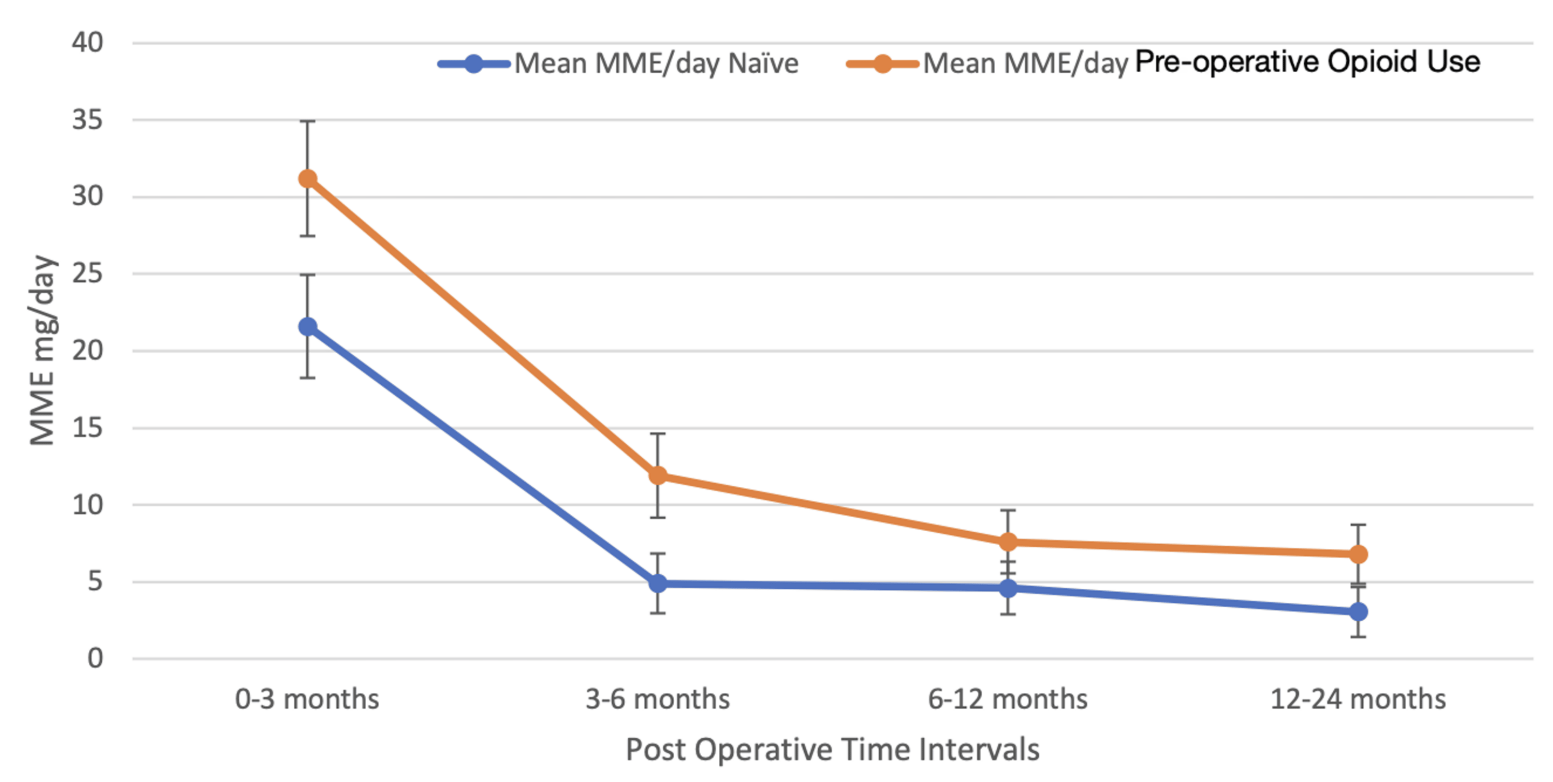
Oral morphine milligram equivalents per day (MME) during specified intervals. Postoperatively, the pre-operative opioid use cohort received significantly higher daily MME compared to the opioid-naïve cohort at 3 months (31.2 mg/d vs. 21.6 mg/d, p=0.011) and 6 months (11.9 mg/d vs. 4.9 mg/d, p<0.001). No difference was found at 12 and 24 months.

In the pre-operative opioid use group, the preoperative average daily MME was 23.5 mg/day. However, this figure increased to 31.2 mg/day at 3 months postoperatively, before gradually declining to 11.9 mg/day at 6 months, 7.6 mg/day at 12 months, and eventually reaching 6.8 mg/day at the 24-month mark. Notably, between the 3-6 month postoperative period, 34 out of 91 patients (37%) were prescribed opioids, a trend that persisted throughout the entire two-year duration, with 33 patients still requiring opioid prescriptions at both the 12 and 24-month intervals, as illustrated in Figure 1.

**Figure 2:**
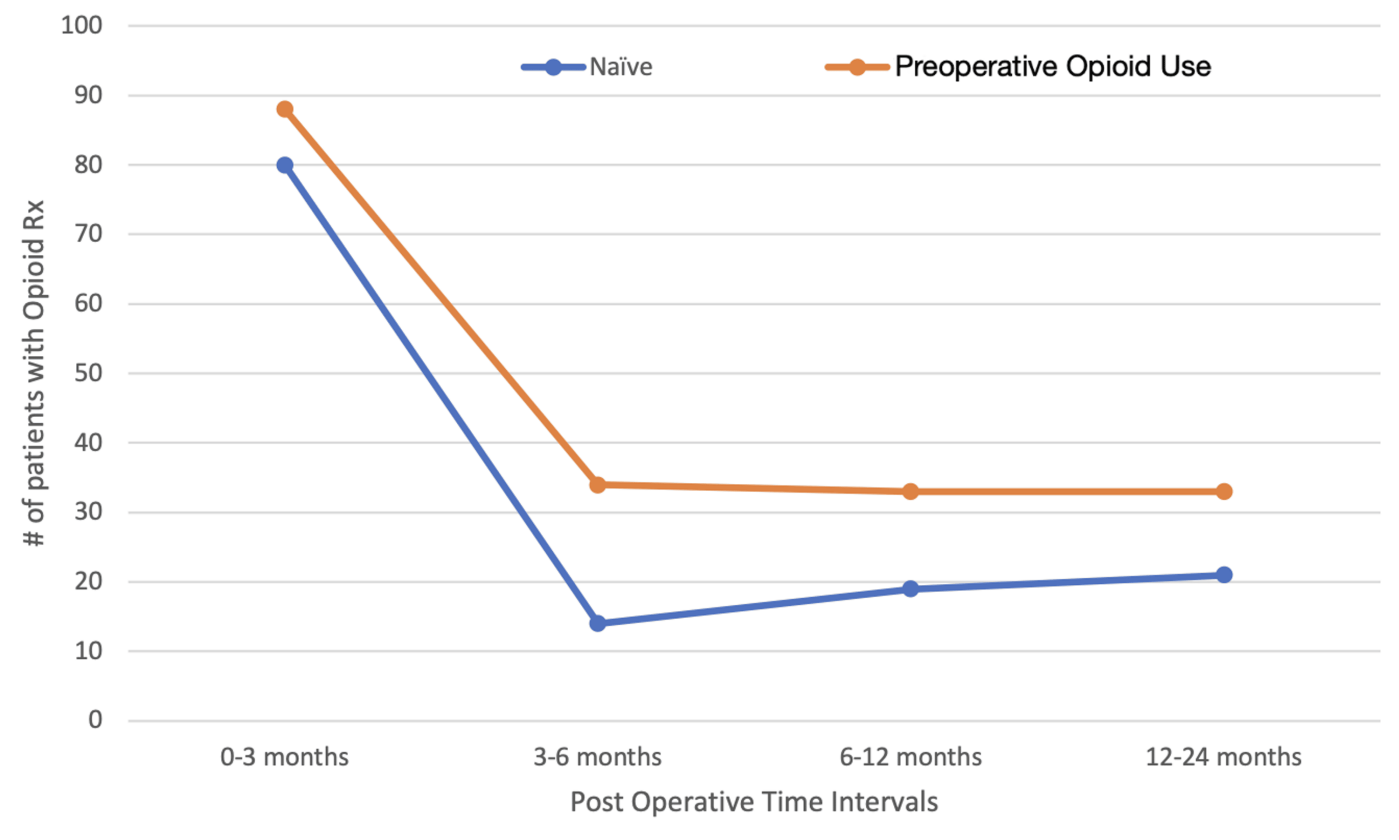
Total number of patients still receiving opioid medications. Between 3 and 6 months, opioid usage decreased in both groups and appeared to plateau from 6 months to 24 months.

Compared to opioid-naïve patients, POU patients exhibited significantly higher daily MME at both 3 months postoperatively, with values of 21.6 mg/day versus 31.2 mg/day (p=0.011), and at 6 months postoperatively, with values of 4.9 mg/day versus 11.9 mg/day (p<0.001). Additionally, POU patients were notably more likely to have an opioid prescription at 6 months postoperatively, with 37% compared to 17% for opioid-naïve patients (p=0.002), and at 12 months postoperatively, with 36.3% compared to 23.2% for opioid-naïve patients (p=0.043), as illustrated in Figure 2.

Furthermore, the pre-operative opioid use group exhibited significantly longer postoperative lengths of stay compared to the opioid-naïve group, with durations of 4.82 days versus 3.78 days, respectively (p=0.004). Moreover, POU patients were notably more likely to be discharged to a skilled nursing facility, with 40.7% of patients in the POU group being discharged to such facilities compared to 18.3% in the opioid-naïve group (p=0.004).

## Discussion

Patients who were prescribed opioids preoperatively exhibited significantly higher postoperative MME requirements in the initial 6 months following surgery and were more likely to require ongoing opioid prescriptions at 6 and 12 months compared to opioid-naïve patients. These findings resonate with earlier investigations into opioid utilization post-primary TKA, such as the seminal study by Zarling BJ et al., which examined a modest cohort of 315 primary total joint arthroplasties [8]. They reported that prescription refills for opioid-naïve patients stood at 22% after one year, escalating to 64% for POU patients. Moreover, the study highlighted varied prescribing patterns, with many patients receiving opioid prescriptions from both their surgeon and subsequent primary care physician.

Similarly, Bedard NA et al. delved into opioid use following TKA in a substantial cohort of over 73,000 patients [9]. Their findings revealed that not only were opioid users at risk of dependence at the one-year mark, but non-opioid users at risk were predominantly females and patients under 50 years of age. Furthermore, prescribing patterns for both opioid users and non-opioid users demonstrated a downward trend to approximately 25% and 10% at 3 months, respectively, and plateaued at 15% and 5% at 6 months.

Our study outcomes for rTKAs closely parallel these findings, with both POU and opioid-naïve groups witnessing a reduction in the number of patients receiving opioids and a corresponding decrease in overall dosage at 3 months, plateauing at 6 months postoperatively. These findings provide valuable guidance for orthopedic surgeons regarding expectations surrounding opioid utilization during the postoperative recovery period following rTKA.

POU patients experienced a significantly prolonged length of stay and were less likely to be discharged directly home, a pattern reminiscent of findings from a study investigating opioid use in primary arthroplasties. This study revealed that patients taking opioids, including tramadol, had an extended length of stay during hospitalization (1.6 vs. 2.0 days) and were twice as likely to be discharged to a skilled nursing facility (6.67% vs. 17.8%), thus necessitating a higher number of episodes of care after surgery compared to opioid-naïve patients [10].

Although our study focused on revision operations, resulting in elevated overall numbers, our observed length of stay (3.8 vs. 4.8 days) and proportion of patients discharged to facilities (18.3% vs. 40.7%) closely mirrored a similar ratio, approximately 25% longer length of stay and twice the number of patients requiring discharge to skilled nursing facilities. These parallels underscore the impact of opioid tolerance on postoperative recovery and discharge disposition, emphasizing the importance of proactive management strategies for this patient population.

One of the notable strengths of this study is its ability to establish a temporal relationship to opioid usage following rTKA. Specifically, the findings indicate a significant decline in opioid use between the 3 and 6-month postoperative periods. This temporal understanding can be invaluable for surgeons when counseling patients about their recovery expectations and the optimal timing for reducing and discontinuing opioid medication.

Moreover, the study sheds light on the trajectory of opioid usage among patients who are POU, or those who have been using opioids for at least 3 months prior to rTKA surgery. Counseling these patients becomes more nuanced, as they may require higher doses initially, but the tapering of dosage is anticipated to commence between postoperative months 3 and 6. By months 6 to 12, the expectation is that two-thirds of these patients will no longer require opioids. This insight provides a roadmap for managing opioid usage in this patient population and setting realistic recovery expectations. Despite these encouraging trends, it is noteworthy that a considerable proportion of patients, both opioid-naïve and POU, continued to receive opioids at the 24-month mark after rTKA surgery. Even though the overall MME trended downward over this period, with the majority of patients experiencing a reduction in opioid dosage, the persistence of opioid use highlights the ongoing challenges in postoperative pain management. This is compounded by the evidence that patients in POU groups had longer lengths of hospitalization and had twice the odds of being discharged to a skilled nursing facility.

One notable limitation of this study is that the prescribing patterns and patient awareness during the study period may differ from current practices. The declaration of the opioid epidemic as a national emergency on October 26, 2017, prompted changes in laws aimed at reducing total opioid prescriptions and increasing patient awareness regarding the negative side effects of opioid medications. Consequently, it is plausible that overall MME and total prescriptions during postoperative recovery may have decreased since then. However, despite these potential changes, our findings still offer valuable insights for surgeons regarding expectations for the timing of opioid reduction and cessation in post-recovery pain management regimens.

Another limitation is that we could only document the prescriptions provided and were unable to record the exact dosage of pain medication consumed by the patient. Therefore, we had to assume that patients who received another prescription had taken all the medication previously provided and were requesting additional medication accordingly. While this represents a limitation, it is a common approach adopted in many opioid studies [2-3, 9].

Lastly, although our institution boasts a reasonably captive patient population (meaning that patients were very unlikely to seek care elsewhere that could go unreported), it is possible that some patients sought opioid prescriptions from other outside institutions, which may not have been captured in our study. This introduces a potential source of bias and underscores the need for caution when generalizing our findings to other patient populations.

Opioid monitoring programs, which vary by state level (e.g., E-FORCSE, CURES, etc.), were not in effect during the study period.

## Conclusions

In summary, this study underscores the impact of preoperative opioid prescriptions on postoperative opioid requirements in patients undergoing rTKA. Those with prior opioid use exhibited higher postoperative opioid needs and were more likely to sustain opioid use at 6 and 12 months postoperatively compared to their opioid-naïve counterparts. However, both groups experienced a decline in opioid utilization from 3 to 6 months postoperatively, followed by stabilization thereafter. Additionally, patients with preoperative opioid use faced prolonged hospital stays and were less likely to be discharged directly home. These findings offer valuable insights for surgeons in managing postoperative opioid expectations and counseling patients throughout their recovery journey.
